# The Use and Abuse of Decinormal Salt Solution

**Published:** 1899-01

**Authors:** 


### Society Reports.

### THE USE AND ABUSE OF DECINORMAL SALT SOLUTION.

From the Proceedings of the Memphis Meeting of the Southern Surgical and Gynecological Association, Dec. 6-8, 1898.

Dr. J. Wesley Bovee, of Washington, D. C., read a paper with the above title.

The term decinormal salt solution had been used interchangeably with artificial serum. Various compositions and strengths of the constituent elements of blood had been used with the name decinormal salt solution. According to Kirkes' "Hand-Book of Physiology," salt existed in the blood plasma in the proportion of 5.546 parts per 1,000, and 6 per cent. was a good practical formula. Other ingredients, i. e., egg albumen, had been added to the salt, which really made an artificial serum instead of decinormal salt solution.

Of the five different routes through which it was introduced, the intra-arterial, suggested by Dawbarn, was considered unsafe in all conditions and should not be practiced. The subcutaneous was the most useful for general use. In emergency work the intravenous method would often be needed in severe hemorrhage, and the rectal enema of the solution would be found of great advantage in nearly all cases in which no bowel lesion was to be combated. In abdominal surgery the peritoneal cavity would be the place selected for its introduction, and even in vaginal opening of the peritoneal cavity, as in hysterectomy from this way, the speaker had thrown considerable quantities into the peritoneal cavity, the hips being elevated at the time and the peritoneal opening closed directly afterward. The intraveneous route, usually the most rapid, might be rendered slower than the subcutaneous by the difficulty of finding a vein and successfully introducing the cannula.

The physiological action of decinormal salt solution was to stimulate powerfully the cardiac ganglia and the nerve centres. The skin, kidney, and intestinal functions were stimulated markedly, and other organs were likewise affected. Osmosis was decidedly promoted by it, and as a result of increased arterial tension the blood supply to the heart muscle was much increased. It had a hæmostatic effect when applied locally to raw surfaces, lessening oozing by stimulating and contracting the smaller vessels. According to Hayem and others, it augmented the number of red blood corpuscles. It was eliminated by the skin, changing the chemical reaction of the perspiration and heavily leading it with salt. The kidneys also carried away an enormous amount of it when large quantities had been introduced into the tissues. The lungs removed it freely, it having been noticed in crystals on the lips after its free use. Autopsies after its use under the skin showed a considerable quantity of it in the intestines, demonstrating that it was thrown off by this route.

The essayist then spoke of its use in general medicine. In surgery the principal indications for its use were shock, hemorrhage, and sepsis. In shock it should be employed early, on the table during operations, or even before the operation had been undertaken in bad cases, or after operation in milder ones. Severe hemorrhage was to be treated in the same manner, though after the flow of blood had been checked. If the hemorrhage was severe, the intravenous route might be employed, it being about the only indication for this method. In the author's abdominal work he almost invariably left a considerable quantity of salt solution in the peritoneal cavity. Its salutary effect was produced by its action on the abdominal viscera with which it came in close contact. To prevent adhesions in the pelvis, one or two litres sufficed. It hastened absorption of stray or concealed blood clots, septic foci, or escaped fluids by carrying them well up into the abdominal cavity in cases of pelvic surgery. In hemorrhage it was probably best to infuse small quantities and often rather than one large quantity.

Judging from the reports of experimenters, the use of decinormal salt solution was not a harmless procedure It was contraindicated in such conditions of the blood as hæmophilia, dyscrasias, deficient fibrin, etc. It would seem not unreasonable that such a strong stimulant coupled with its dilatation of the blood-vessels when used in large quantities, and specially when thrown directly into them, would be very harmful in such conditions of the circulatory apparatus as myocarditis, pericardial effusion, atheroma, arteriosclerosis, cardiac degeneration, bad valvular lesions, thrombosis, and recent cerebral apoplexies. Chronic diseases of the lungs, kidneys, or liver, especially if malignant, were apt to be aggravated by it. Active hemorrhage in any location was aggravated by it. The presence of toxins in the blood had been found to retard the elimination of decinormal salt solution, and, for that reason, only small quantities at a time should be employed.

It was necessary to avoid certain accidents and mistakes in using decinormal salt solution. The solution must be known to be sterile when it entered the tissues of the body, except when injected by the rectum, in which case sterility was of no moment. Air-bubbles should not be allowed to enter into blood-vessels or cellular tissue. The fluid must be of a sufficiently high temperature when reaching the body. Chills occurred from a cold solution and were dangerous to very weak patients. The vessel containing the solution, as well as the tube and needle conducting it, must be aseptic and thoroughly pervious; the tube shou d have a glass window that the rapidity of the current and the presence of any foreign body could be noted. When the solution was to be introduced through the skin, either into cellular tissue or vein, the local surface should be cleaned as much as the limited time would permit. Probably not more than a half litre should be injected into the tissue through one puncture, as localized necrosis and a septic inflammation have resulted from over-distention of the tissue spaces. Ordinarily not more than one ounce per minute should be injected into tissue or vein. Pulmonary œdema, dyspnœa, headache, vertigo, mental excitement, delirium, hallucinations, and severe pain in the left side occurred from over-distention of the veins from too large an infusion.

In the discussion Drs. Parker, Johnson, Noble, Parham, Cartledge, Tiffany, Kelly, McMurtry, Baxter, McRae and Brown spoke favorably of the use of decinormal salt solution.

Dr. Willis F. Westmoreland, of Atlanta, presented a paper entitled

### CARCINOMA OF THE BREAST.

He said that carcinoma now presents even a more serious problem than formerly, on account of its rapid increase. The proportionate mortality from cancer is four and a half times greater now than half a century ago. No other disease can show anything like such an immense increase. According to statistics, two out of every five cases of carcinoma in the female are of the breast. Over three-fourths of all the tumors occurring in the breast are carcinoma, or, to be exact, of 627 tumors, over 503, or 83.20 per cent. were carcinoma, leaving only 107, or 16.79 per cent., representing all other forms of neoplasms. The author discussed the etiology of the disease at considerable length. Speaking of the pathology, he said there was no subject in surgery about which such divergent opinions are held. There is a wide variation in the malignancy of carcinoma, some proving fatal in less than a year, a few others taking ten or fifteen years. He divided the new varieties of carcinoma into the scirrhous and medullary or encephaloid. The former has an average duration of life of about thirty months, the latter of about twelve months. When it is considered that the skin and pectoral muscles are involved in nearly every instance in this disease, the surgeon can easily see the philosophic reasoning upon which Halstead's operation is based. The essayist follows the Halstead method in carcinoma of the breast. He believes, however, that Dr. Bloodgood was the first to demonstrate the advantages of completely cleaning out the posterointernal subscapular region by the supraclavicular route. The results of the Halstead operation, in the opinion of the essayist, depend upon the ability of the individual operator, his earliness in seeing the case and his closeness in following Halstead's technique. This operation not only requires surgical skill and experience, but proper respect for the magnitude of small things.

In the opinion of Dr. Westmoreland, no surgeon will make a satisfactory operation on his first few cases. He finds that with each case he operatees more satisfactorily. The operation does not present the seriousness that its magnitude would lead us to believe. He has operated six times before his class at a public clinic and transferred the patients afterward, at times quite a distance, to their homes. In his first operations he had some shock from exposure, but this is easily controlled by placing hot towels over the exposed surfaces. There is practically no blood lost in a properly-made operation. It is better to complete the operation, grafting included, at one sitting. When, for any reason, this is not practicable, the cleaning out of the supraclavicular region and the grafting can be made at a second operation. When grafting is done at a second operation he finds it better to remove the granulations. This is best done by curetting. In two of his cases it was necessary to remove the subscapular nerves, and in another to resect the axillary vein. No unpleasant sequelæ followed in either case. He has operated upon over fifty cases of carcinoma since he began surgical work, and has always cleansed out the axilla and stripped the muscles in each case. He has been making Halstead's operation since its introduction. He does not know the results in the majority of these cases. Two of them, however, came back to him with later recurrence, one with a carcinoma of the femur five years after the operation, no local return; the other with a local return in the cicatrix nine years afterward. This case died in a short time from metastasis. He feels quite sure that these were not independent new growths. There is always one rule, that the more malignant the case, the greater the blood changes.

The primary object of the paper was to point out the fact that a careful examination of each case, a record of the local conditions, the type of tumor, the blood changes, whether or not there was marrow infection, the family history, and the length of time it had existed, would not only give definite facts upon which to base a prognosis, but, if published with each case, would give all surgeons a record of infinite value for comparative purposes.

Dr. F. W. Parham, of New Orleans, read a paper on

RESECTION OF THE THORACIC WALL FOR TUMORS GROWING FROM THE THORACIC SKELETON.

He reported two such operations of his own with successful results. Both operations widely opened the plural cavity and the lung collapsed in both. In the first the collapse was relieved only when the great pectoral muscle was brought down and sutured to the rent in the pleura. In the other, signal service was rendered by the Fell-O'Dwyer apparatus for maintaining regular respiration. He believes the perfection of this apparatus and its application in skillful hands will greatly aid in the developing of the surgery of the chest. He had collected all cases operated on up to date with a mortality of 25 per cent. for the intraplural operations for tumors of the chest-wall.

Dr. John T. Wilson, of Sherman, Texas, read a paper on

FRACTURES INVOLVING THE ELBOW-JOINT.

He said: Fractures involving the elbow-joint are of great interest and always cause more or less anxiety because of their liability to ankylosis, partial or complete; to deformity, or to some interference with the perfect function of the joint as a result. Fractures without the capsular ligament are not so grave, are under better control, give more satisfactory results, and cause less apprehension. Fractures within the ligaments are always troublesome, give rise to more inflammation, demand greater care in their reduction, in the dressing, and in the after-treatment. Ankylosis in various degrees is generally looked for when union has taken place; it is extremely difficult in many cases to avoid this result, but with care, patience and perseverance on the part of the surgeon and patient, this complication can be remedied wholly or in part in nearly all cases. It is necessary to remember that the muscular attachments to the region around the joint are very great. It is difficult to conceive of a fracture here where the fragments do not involve a muscular attachment, and are more or less displaced by it. A knowledge of these attachments is sufficient to indicate the difficulty of confining the broken bones in perfect apposition. In fractures of this point there is usually considerable inflammation ensuing, the cartilage of the articular surfaces being wounded and the investing ligaments lacerated. The amount of callus thrown out is considerable; it hinders motion, and is generally slow of absorption.

To get the best practical results in these fractures, it is of supreme importance to keep the parts at absolute rest. To do this, the joint must be put up in a suitable fixation dressing and patient kept in quiescent state; in order to do this it is necessary to confine patient to bed for a week or more, and a fixation dressing is applied in the beginning of the treatment. Eminent surgeons differ in regard to the position of the fore-arm. The different fractures and the different degrees of fracture require different positions of the fore-arm, require different positions in pronation, supination and the angles. Either extreme strikes him as being irrational. He is guided by that position which favors best the coaptation of the fragments and the relaxation of the muscles, giving the least deformity, and the flexed position in some degree has been the one usually employed. After an aseptic scrubbing of the joint, arm and fore-arm under an anesthetic the diagnosis is made, if possible, and the dressings applied. After a nice application of the spiral reversed roller, not too tight, a firm pad of sterilized gauze is placed in front of the joint opposite the broken fragments, thick enough for a firm support, and not as thick as to interfere with the functions of the splint. The anterior splint is then put in place, one with a ratched hinge much like that of Dr. Agnew, which can be placed at any angle desired and extending from near the wrist to above the insertion of the deltoid. A pasteboard trough is then moulded to the posterior surface of the limb extending from the axilla to the ends of the fingers and confined by a roller. If no contraindication the dressing is not disturbed for three or four days, or longer, if deemed advisable; it is then carefully removed, the joint examined, and if the inflammation has subsided the permanent dressing is applied.

The correct position is maintained by the anterior splint and a carefully moulded plaster of paris trough is fitted posteriorly; this must be adjusted nicely in order to support the fragments, keeping them in place posteriorly and laterally. This should also extend from the axilla and support the hand, should not be heavy and cumbersome, but firm enough to thoroughly fix the joint and arm. This can be done by having some strips of heavy gauze or cheese-cloth measured by the sound arm and carefully cut to the proper size; the plaster is thoroughly rubbed in these and they are then applied, the arm being well protected, layer by layer, a solution of plaster rubbed in between each layer until of the thickness desired. This dressing can be controlled at will, and if nicely applied is not so cumbersome as it would seem. If it becomes desirable to change the position or angle, the plaster trough can be taken off by simply removing the roller, the position changed and a new trough applied. He prefers to have the anterior splint of wood broad enough to cover the joint, and yet not extending quite to the ends of the epicondyles, the joint fitting closely down to the bend of the elbow, the ratched notches changing not more than the eighth of an inch at each movement. If properly made, this splint is light enough and yet firm enough for all practical purposes, and much of its usefulness depends upon its fitting well, and so with the plaster trough. If extension and counter-extension are necessary, these can be had by extending the plaster trough under the axilla and the anterior splint will make sufficient counter-pressure by means of the roller. This gives gives a firm, fixed dressing, keeping the parts in place and at rest, so necessary in all inflammatory conditions of the joints. The patient is seen frequently, the joint examined at any time when there seems to be an indication for it; this should never be done, however, if there is no special indication for it after the permanent dressing has been applied and the surgeon satisfied that the fragments are in place. If kept in this position and joint at complete rest, danger of ankylosis is not great.

After three or four weeks, not earlier unless there is special indication for it, dressing can be renewed, the joint examined with care and, if any stiffness, passive motion gently instituted under an anesthetic, if necessary. If only fibrous bands exist, they can be broken up. Violence should not be used. Gentle motion, frequently repeated and persevered in, will often accomplish more than sudden forcible separation with shock attending it. Rubbing and systematic massage at this stage is of great value, and if stiffness is not readily overcome, persistence in the gentle efforts continued; after five or six weeks patient is instructed to gently exercise the joint twice daily with Indian clubs and dumb-bells, neither of which should be too heavy. He is advised to use the hand and arm constantly, and in time, in the great majority of cases, will be rewarded with success. In some cases improvement may take many months or two or three years before the function is restored. If there are several fragments of bone and some so small they can not be kept in apposition, their removal would save time and trouble and give a more favorable chance for a better joint. If ankylosis has supervened upon a fracture and many months of patient effort at restoring the function of the joint proved futile, an operation is justified. If a fragment is causing the trouble, it can be removed. A resection will often give a good useful joint.

(We owe the above to the *Alabama Medical and Surgical Age*.)

---

The hunter rose at break of morn,
  When the cold winds blew infernally,
He took his frisking dog and gun
  And took a horn—internally.

